# Biochemical Sensors for Personalized Therapy in Parkinson’s Disease: Where We Stand

**DOI:** 10.3390/jcm13237458

**Published:** 2024-12-07

**Authors:** Davide Ciarrocchi, Pasquale Maria Pecoraro, Alessandro Zompanti, Giorgio Pennazza, Marco Santonico, Lazzaro di Biase

**Affiliations:** 1 Unit of Electronics for Sensor Systems, Department of Engineering, Università Campus Bio-Medico di Roma, 00128 Rome, Italy; davide.ciarrocchi@unicampus.it (D.C.); a.zompanti@unicampus.it (A.Z.); g.pennazza@unicampus.it (G.P.); 2 Operative Research Unit of Neurology, Fondazione Policlinico Universitario Campus Bio-Medico, Via Álvaro del Portillo, 200, 00128 Rome, Italy; p.pecoraro@unicampus.it; 3 Research Unit of Neurology, Neurophysiology and Neurobiology, Department of Medicine and Surgery, Università Campus Bio-Medico di Roma, Via Alvaro del Portillo, 21, 00128 Rome, Italy; 4 Unit of Electronics for Sensor Systems, Department of Science and Technology for Sustainable Development and One Health, Università Campus Bio-Medico di Roma, 00128 Rome, Italy; m.santonico@unicampus.it; 5 Brain Innovations Lab, Università Campus Bio-Medico di Roma, Via Álvaro del Portillo, 21, 00128 Rome, Italy

**Keywords:** Parkinson’s disease (PD), biochemical sensors, levodopa, closed-loop therapy

## Abstract

Since its first introduction, levodopa has remained the cornerstone treatment for Parkinson’s disease. However, as the disease advances, the therapeutic window for levodopa narrows, leading to motor complications like fluctuations and dyskinesias. Clinicians face challenges in optimizing daily therapeutic regimens, particularly in advanced stages, due to the lack of quantitative biomarkers for continuous motor monitoring. Biochemical sensing of levodopa offers a promising approach for real-time therapeutic feedback, potentially sustaining an optimal motor state throughout the day. These sensors vary in invasiveness, encompassing techniques like microdialysis, electrochemical non-enzymatic sensing, and enzymatic approaches. Electrochemical sensing, including wearable solutions that utilize reverse iontophoresis and microneedles, is notable for its potential in non-invasive or minimally invasive monitoring. Point-of-care devices and standard electrochemical cells demonstrate superior performance compared to wearable solutions; however, this comes at the cost of wearability. As a result, they are better suited for clinical use. The integration of nanomaterials such as carbon nanotubes, metal–organic frameworks, and graphene has significantly enhanced sensor sensitivity, selectivity, and detection performance. This framework paves the way for accurate, continuous monitoring of levodopa and its metabolites in biofluids such as sweat and interstitial fluid, aiding real-time motor performance assessment in Parkinson’s disease. This review highlights recent advancements in biochemical sensing for levodopa and catecholamine monitoring, exploring emerging technologies and their potential role in developing closed-loop therapy for Parkinson’s disease.

## 1. Introduction

Despite various refinements in therapeutic strategies and add-on options, levodopa is still the mainstay for the treatment of Parkinson’s disease (PD) since its first introduction in 1969 [1].

**Figure 1:**
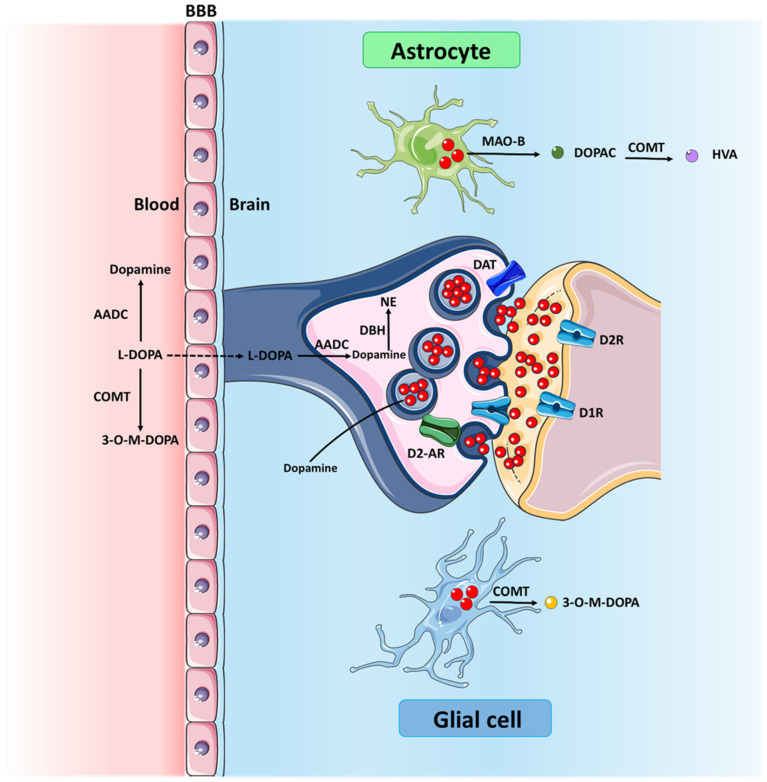
Synaptic metabolic pathways of levodopa. Abbreviations: AADC = aromatic L-amino acid decarboxylase; L-DOPA = levodopa; COMT = catechol-O-methyltransferase; 3-O-M-DOPA = 3-O-Methyldopa; DBH = Dopamine-β-hydroxylase; DAT = dopamine transporter; DR = dopamine receptor; D2-AR = dopamine autoreceptor; DOPAC = 3,4-Dihydroxyphenylacetic acid; MAO-B = monoamine oxidase-B; NE = norepinephrine; HVA = homovanilic acid.

After oral intake, the primary metabolic fate of levodopa is its decarboxylation to dopamine, catalyzed by the enzyme aromatic L-amino acid decarboxylase (AADC). This enzyme is found throughout the body, including the peripheral tissues and the central nervous system (CNS). The conversion of levodopa to dopamine represents the critical step in making the active catecholamine neurotransmitter available for synaptic transmission and other physiological effects [2]. Dopamine itself then undergoes further metabolism through two main enzymatic pathways in astrocytes and glial cells: methylation by catechol-O-methyltransferase (COMT) to 3-O-Methyldopa (3-OMD) and oxidative deamination by monoamine oxidase (MAO), primarily the MAO-B isoform, to 3,4-dihydroxyphenylacetic acid (DOPAC). DOPAC can then be further metabolized by COMT to homovanillic acid (HVA) [3]. In this context, the balance between MAO and COMT pathways regulates the overall catabolism and clearance of dopamine, indirectly influencing the patient’s motor state. Figure 1 depicts the main synaptic and intracellular levodopa metabolic pathways.

An in-depth understanding of the transport kinetics of dopamine metabolites across the blood–brain barrier (BBB) is pivotal for optimizing levodopa treatment efficacy and minimizing drug-induced side effects in PD. The BBB serves as a critical regulatory interface, restricting the passage of most molecules while facilitating the selective transport of levodopa and its metabolite 3-OMD, via the large neutral amino acid transporter 1 (LAT-1) [4]. This transporter, which operates under a saturable mechanism, mediates competition among substrates, meaning that elevated plasma levels of 3-OMD can impair the efficient uptake of levodopa into the CNS. This phenomenon can lead to suboptimal neuronal levodopa availability, preventing clinical response and exacerbating motor fluctuations [5].

Nonetheless, the kinetics of metabolite transport, including rates of influx and efflux, influences not only levodopa efficacy but also the potential accumulation of byproducts such as 3-OMD, which may exert neurotoxic or neuroadaptive effects. For instance, 3-OMD competes with levodopa and other neutral amino acids for LAT-1-mediated entry into the brain, potentially reducing the bioavailability of levodopa for conversion into dopamine in striatal neurons [6,7]. This competitive inhibition is particularly relevant in advanced PD, where the therapeutic window narrows, and motor complications such as dyskinesias are more prevalent [5,8]. In contrast, the transport of dopamine’s downstream metabolites, e.g., DOPAC and HVA, primarily reflects efflux dynamics and the overall turnover of dopamine within the brain [9]. These metabolites are transported out of the CNS into the systemic circulation for renal excretion. Efflux transporters, such as the multidrug resistance-associated protein (MRP) family, play a role in this clearance process, and the dysregulation of these pathways could affect metabolite accumulation, further influencing CNS dopamine homeostasis and bioavailability [10].

These insights underscore the importance of considering BBB dynamics in optimizing treatment efficacy and mitigating the challenges of long-term levodopa therapy. For example, the pharmacological modulation of COMT to reduce peripheral 3-OMD levels or LAT-1 substrate competition could enhance levodopa delivery to the CNS [5]. Similarly, targeting efflux transporters involved in DOPAC and HVA clearance might provide additional therapeutic leverage by modulating dopamine turnover.

Importantly, dopamine is the precursor for the synthesis of both norepinephrine and epinephrine. The dopamine β-hydroxylase converts dopamine into norepinephrine by adding a hydroxyl group. Norepinephrine can then be further methylated by the enzyme phenylethanolamine N-methyltransferase to produce epinephrine. This biosynthetic pathway links the three catecholamines together, as dopamine availability influences the levels of norepinephrine and epinephrine.

**Figure 2:**
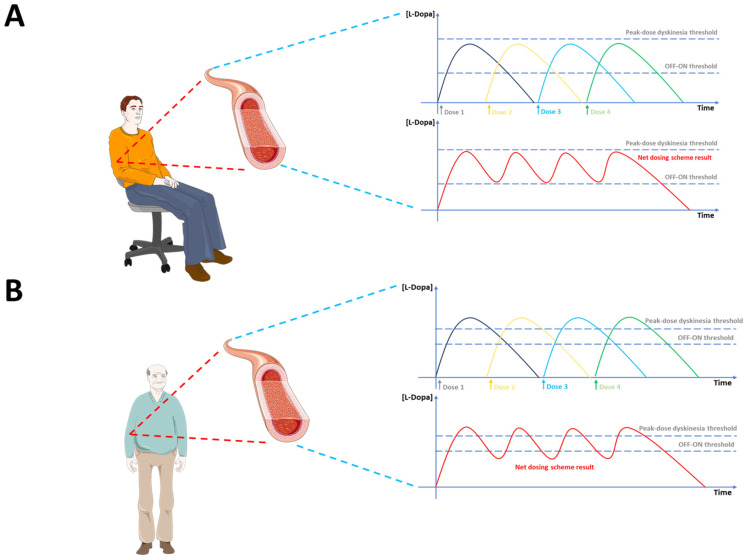
Pharmacokinetic profiles of single doses of levodopa for early-stage PD (**A**) and advanced-stage PD (**B**). Top row (**A**): In early-stage PD, the pharmacokinetic profile of each levodopa dose (Dose 1 to 4) shows a gradual rise and fall in plasma levels. The concentration remains consistently between the OFF–ON transition threshold (lower dashed line) and the peak-dose dyskinesia threshold (upper dashed line), ensuring effective symptom control without inducing peak-dose or biphasic dyskinesias. The lower-right graph in this row represents the combined net effect of the four doses, showing a controlled and steady plasma levodopa concentration within the therapeutic range. Bottom row (**B**): In advanced-stage PD, while the pharmacokinetic behavior of each single dose (Dose 1 to 4) appears preserved, significant pharmacodynamic changes lead to a narrowing of the therapeutic window. The plasma concentrations more frequently exceed the peak-dose dyskinesia threshold, causing dyskinesias, and drop below the OFF–ON threshold, resulting in motor fluctuations such as “wearing-off”, “No-ON”, or “delayed ON” periods. The lower-right graph in this row illustrates the combined net effect of the four doses, characterized by increased variability and greater difficulty maintaining plasma levels within the therapeutic range. Legend: Lower dashed line: Plasma levodopa level [L-Dopa] threshold for OFF–ON transition (symptom improvement). Upper dashed line: Plasma levodopa level [L-Dopa] threshold for peak-dose dyskinesia (motor complications).

During PD progression, the levodopa therapeutic window narrows, the threshold for peak-dose dyskinesias decreases, and motor complications like levodopa-induced dyskinesias (LIDs) and motor fluctuations subsequently arise [11], as shown in Figure 2. In this context, the primary distinction between stable and fluctuating PD patients appears to stem from pharmacodynamic changes occurring throughout the disease progression, rather than pharmacokinetic factors [12,13,14].

**Figure 3:**
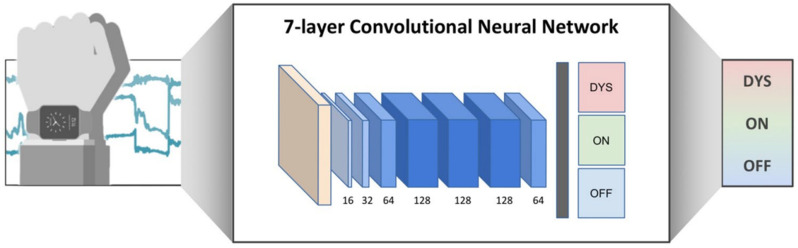
Configuration of IMU sensor data collection and implementation of a seven-layer convolutional neural network model. Figure drawn by Dr. Pfister, and reproduced, under the terms of the Creative Commons Attribution 4.0 License, from [36].

As a consequence, drawing up a daily antiparkinsonian therapeutic regimen that balances OFF time and LIDs in advanced PD patients is challenging for the clinician, mainly due to the absence of quantitative objective biomarkers for motor performance monitoring. Administering the dose of levodopa effectively needed by the motor state of the patient is an unmet need with the current standard oral therapeutic fixed-dosage scheme [15,16]. Nowadays, the clinical evaluation of motor performances remains the gold standard for a gross estimation of plasma levodopa level in routine practice. However, the phenomenology of PD and other movement disorders is often challenging to accurately assess solely through clinical examination: most clinical scales adopted to assess disease severity suffer from poor interrater reliability, high inconsistency, and limited intraclass coefficients [17]. In this regard, new technologies like wearable motion sensor devices integrated with neurophysiological tools like Electroencephalogram (EEG) have been paving the way not only for continuous monitoring of symptoms at home [18] but also for the quantitative characterization of the whole spectrum of PD motor symptoms [19,20,21,22,23], like tremor [24,25,26,27,28,29], bradykinesia [30], rigidity [31,32], gait, balance, and postural issues [33,34], alongside motor complications like motor fluctuations, dyskinesias, and dystonia [35,36,37,38,39]. In addition, data science with the development of artificial intelligence and machine learning (ML) algorithms will further improve both motor symptom identification [40,41,42] and therapeutic regimen optimization to avoid motor complications [43], not only in PD but also in the stroke field [44,45,46]. For this purpose, Pfister et al. [36] classified motion data, recorded with a single wrist-worn inertial measurement unit (IMU), through a deep learning model in a free-living setting, as summarized in Figure 3.

### Biochemical Sensing in Parkinson’s Disease

Adaptive and closed-loop-based strategies have been addressed for the management of motor complications in PD [47] to overcome conventional continuous pharmacological/electrical stimulation paradigms [48,49,50,51,52,53]. These innovative methods dynamically adjust therapeutic regimens in real time, leveraging biomarker-driven feedback to ensure optimal motor performance is maintained throughout the entire day. A key aspect of this approach is identifying robust biomarkers that reliably correlate with and are sensitive to variations in motor states. In this regard, biochemical sensing has emerged under the spotlight in PD for its reduced invasiveness and variety of approaches, ranging from microdialysis [54,55,56] to electrochemical sensing techniques [57,58,59].

Catecholamines such as levodopa in the presence of different compounds can be detected by modifying electrodes with several advanced nanomaterial groups, such as polymers, metal oxide nanoparticles, carbon nanotubes, and graphene oxide, even when strong interferences like ascorbic acid and uric acid are present.

Dopamine and catecholamine levels have a variable range depending on age, ambulatory or supine condition, and substrate (plasma, urine, or sweat) and are usually measured in the laboratory using high-performance liquid chromatography (HPLC) or mass spectrometry. The plasma dopamine level is generally <60 pg/mL [60], while urinary levels can reach 400 mcg/24 h [61]. The physiologic range for norepinephrine is 70–1700 pg/mL, while for epinephrine, it is up to 140 pg/mL [62,63].

Monitoring the levels and ratios of levodopa, dopamine, norepinephrine, epinephrine, DOPAC, and HVA has the potential to provide insights into the underlying biochemical processes that influence the motor state in PD, but it is not as easy to pursue since those metabolites are influenced by numerous factors. In this regard, dopamine and catecholamine levels demonstrate significant variability due to age, posture (ambulatory or supine), and biological sampling substrates (plasma, urine, or sweat) [62,63]. Nonetheless, the fasting state profoundly impacts catecholamine metabolism: fasting activates the sympathetic nervous system, leading to increased norepinephrine and epinephrine levels as part of the physiological adaptation to nutrient deprivation [64]. This is mediated through enhanced tyrosine hydroxylase activity, which catalyzes the rate-limiting step in catecholamine biosynthesis. In contrast, dopamine levels may decrease during fasting due to the reduced availability of precursors like tyrosine and the metabolic shift toward gluconeogenesis and lipid metabolism [64]. It is important to note that the pharmacokinetics of orally administered levodopa are influenced by gastric emptying, as absorption occurs exclusively in the proximal one-third of the small intestine and not in the stomach. Gastric motility varies between fed and fasted states, and erratic gastric emptying introduces unpredictability to the levodopa concentration–time curve. Furthermore, gastric emptying time is often delayed in advanced PD patients, particularly those experiencing motor fluctuations [6,65,66,67]. These changes alter dopamine degradation pathways, increasing oxidative stress within catecholaminergic neurons due to reactive oxygen species (ROS) generated during dopamine metabolism [64]. This metabolic reprogramming underscores the importance of considering fasting status in both clinical and research settings to accurately interpret dopamine and catecholamine measurements. Such insights can help to refine diagnostic and therapeutic strategies in advanced-stage PD, where dopamine homeostasis is critical: integrated models should take all the mentioned factors into account for reliable and adaptable biochemical sensing.

Biochemical sensors can take advantage of various sensing methods, directly from sweat [68,69] on the skin, blood [70], subcutaneous tissue [56], or skeletal muscles [71]. These new methods monitor the pharmacokinetic and pharmacodynamic behavior of levodopa and its metabolites.

Levodopa sensing relies on mathematical models of levodopa pharmacokinetics and pharmacodynamics, designed to predict motor responses based on plasma concentration levels [13,72]. Baston et al. [13] proposed neuro-computational models capable of predicting levodopa plasma concentrations over time, estimating brain levodopa levels as a function of plasma concentrations and modeling the resulting temporal patterns of finger-tapping frequency [73]. An advanced iteration of this model demonstrated a high correlation with real data in predicting both levodopa plasma concentrations and the temporal patterns of finger-tapping motor responses in stable and fluctuating PD patients [72].

**Figure 4:**
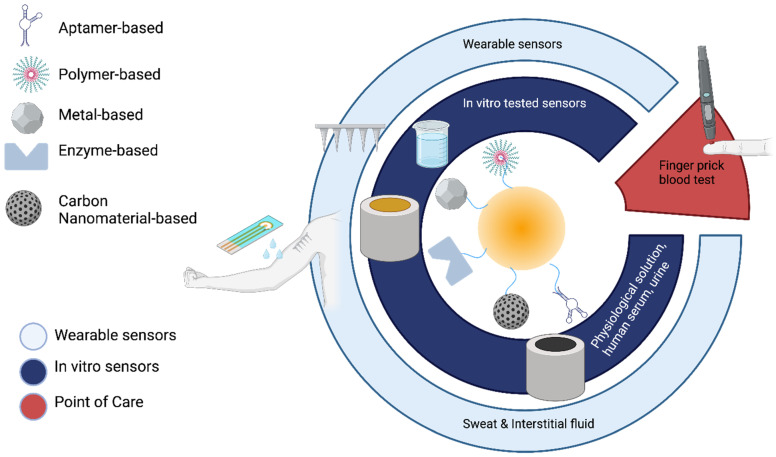
Schematic diagram illustrating biosensors for catecholamine detection, differentiated by surface modification. The sensors are categorized based on their applicability: minimally invasive, tested in vitro, and portable. Additionally, they are classified according to the type of sample analyzed.

Electrochemical methods for catecholamine detection can be categorized based on invasiveness, the type of sample analyzed, and the technological platform employed. Biochemical sensing techniques primarily include microdialysis, which measures absolute concentrations of specific molecules, and electrochemical sensing (amperometry or voltammetry), which tracks relative concentration changes with higher temporal and spatial resolution. These methods span non-invasive and minimally invasive wearable technologies like reverse iontophoresis, microneedles, and sweat sensors, as well as point-of-care solutions such as dried blood spot (DBS) analysis. Invasive approaches like microdialysis and conventional electrochemical cells are also employed. Furthermore, advanced nanomaterials—such as graphene, carbon nanotubes, and metal–organic frameworks—have been integrated to enhance the sensitivity and selectivity of catecholamine monitoring across all these platforms. Figure 4 graphically summarizes the classification of biochemical sensors for catecholamine detection.

In this review, we examine recent advancements in catecholamine sensing over the past decade, with a particular focus on the innovative developments introduced by nanomaterials. We begin by classifying and summarizing electrochemical sensors based on their wearability, as illustrated in Figure 4. Subsequently, we describe the latest technologies leveraging nanomaterials for the development of non-invasive or minimally invasive sensors. Additionally, the work includes a dedicated section on dried blood spot sensors, an emerging and practical option for real-time sensing through autonomous blood sampling.

The primary aim of this descriptive review is to explore the characteristics and biochemical mechanisms underlying non-invasive levodopa and catecholamine sensing models in blood, highlighting their potential to augment motor performance monitoring in fluctuating PD patients for routine clinical practice.

## 2. Wearable: Non-Invasive and Minimally Invasive

**Figure 5:**
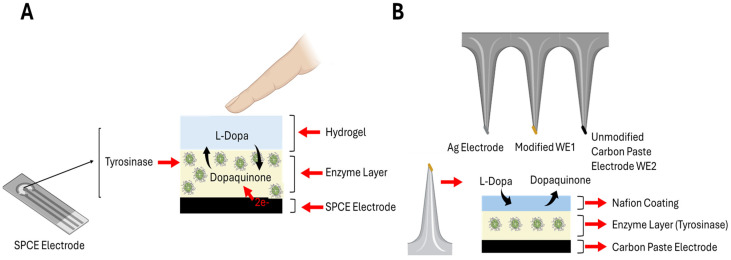
Working process of two portable and wearable biosensor examples for L-Dopa sensing. (**A**) An SPCE with enzyme functionalization and hydrogel as the exchange interface for the biological fluid to be analyzed. The enzymatic reaction occurs on aSPCE modified with tyrosinase, where L-Dopa is oxidized to a dopaquinone layer, releasing two electrons (2e^−^). The hydrogel layer plays a crucial role in facilitating the diffusion of L-Dopa to the electrode surface while maintaining a hydrated environment for the enzymatic reaction. (**B**) Enzyme-based microneedles with Nafion coating as antifouling layer. The microneedle tips are coated with a carbon paste electrode and modified with an enzyme layer of tyrosinase for the detection of L-Dopa. A Nafion coating is applied to enhance selectivity and stability. The enzyme catalyzes the oxidation of L-Dopa to dopaquinone, releasing electrons (2e^−^), which are detected by the underlying electrode, generating an electrochemical signal for real-time monitoring. Abbreviations: SPCE = screen-printed carbon electrode; L-Dopa = Levodopa.

Tai, Liaw, Lin, Nyein, Bariya, Ji, Hettick, Zhao, Zhao, Hou, Yuan, Fan, and Javey [68] developed a skin patch for non-invasive sweat analysis through reverse iontophoresis for extraction and a three-electrode patch with a tyrosinase-functionalized electrode for levodopa detection. This patch took advantage of thionine to obtain the electropolymerized form of polythionine through electrodeposition and a layer of Nafion resin to ensure stability and prevent enzyme leakage. The sensitivity of the device was reported to be 1.7 nA/µM within a linear range of 1.25 µM to 20 µM, with a limit of detection of 1.25 µM. Moon, et al. [74] proposed an enzymatic approach for non-invasive sweat measurement to investigate the pharmacokinetics of levodopa after oral administration. The sensor used a three-electrode system with carbon paste electrodes coated with a layer of hydrogel matrix containing tyrosinase enzyme, allowing real-time monitoring of levodopa pharmacokinetics. The reported linear range was 5 to 30 µM with a detection limit of 300 nM. Figure 5 shows a schematic representation of a screen-printed carbon electrode (SPCE)-based electrochemical sensor (A) and a microneedle-based electrochemical sensor (B) for levodopa sensing.

Xiao, et al. [75] proposed a wearable, non-invasive, and portable electrochemical sensor based on a metal–organic framework (MOF) with an integrated enzyme to monitor the sweat concentration of levodopa. The researchers prepared Zeolitic imidazolate framework/graphene oxide (ZIF-8/GO) composites by growing ZIF-8 nanoparticles on the surface of GO and then loading tyrosinase onto the composite for the scope. The sensor demonstrated a broad linear response ranging from 1–95 µM, a low detection limit of 0.45 µM, and high selectivity toward the target analyte. Furthermore, K.P.O, et al. [76] described an innovative sensor for dopamine using acid-treated carbon cloth (ACC)-alpha-Fe_2_O_3_ nanoparticles on carbon cloth. The proposed sensor achieved a low limit of detection of 50 nM and a linear range of 0.074–113 µM.

Fang, et al. [77] presented a highly innovative system based on an array of flexible differential microneedles to detect levodopa. The microneedles interacted with interstitial fluid, providing a superior alternative to reverse iontophoresis. The resulting sensor comprised two working electrodes, each with specific layers: WE1 and WE2. WE1 is composed of an Au nanodendrite catalytic layer, a Nafion layer, a PANI/enzyme layer, and a polyurethane (PU) layer. WE2 is composed of the same layers but without the PANI/enzyme layer. The sensor exhibited broad linearity (0–20 µM), high sensitivity (12.618 nA/µM), and a lifespan of two weeks. In comparison, Goud, et al. [78] proposed a system based on hollow microneedles of carbon pastes modified with tyrosinase enzyme. This system demonstrated good linearity (5–100 µM) and sensitivity (0.037 µA/µM) and a detection limit of 0.5 µM. Finally, Park, et al. [79] proposed a different approach, based on an array of interdigitated electrodes (IDE) integrated with swellable micro-needles. This combination offered rapid sampling, minimizing tissue damage. Performance features included significant linearity (1–1000 µM) and a detection limit of 1 µM.

Table 1 summarizes wearable bio-sensor performances for catecholamine detection.

Concerning continuous biochemical sensing, reverse iontophoresis and microneedle-based sensors differ mainly in sample acquisition. Indeed, reverse iontophoresis utilizes a small electric current to extract molecules through sweat glands [87], providing access to sweat whose composition can be influenced by factors such as hydration, physical activity, and emotional state [88]. However, the use of an electrical current may lead to skin irritation, posing challenges for long-term monitoring. Microneedle sensors penetrate the stratum corneum to access interstitial fluid (ISF), which closely reflects blood biochemistry [89]. This direct access enables more accurate and timely measurements with minimal lag. Although they create microscopic punctures in the skin, microneedle sensors are significantly less discomforting than traditional subcutaneous sensors.

The sampling method plays a crucial role in ensuring measurement reliability. While reverse iontophoresis is non-invasive, its variable extraction efficiency necessitates frequent calibration. In contrast, microneedle sensors provide more consistent measurements; however, they require periodic replacement due to biofouling and sensor degradation.

## 3. Point of Care (POC): Dried Blood Spot

Dried blood spot (DBS) technology enables easy and non-invasive sample collection by merely spotting a small bit of blood onto filter paper, removing the need for venipunctures and requiring only a limited blood sample amount. DBS samples can even be collected and stored at room temperature due to their stability for more than 28 days at 37 °C, ensuring the integrity of the sample during transportation and analysis and making it convenient for sample transportation and storage. The stability of biomaterial deposited on membrane-based carriers in dried form is essential, requiring no specialized storage protocols, unlike cellular specimens or plasma samples [90].

**Figure 6:**
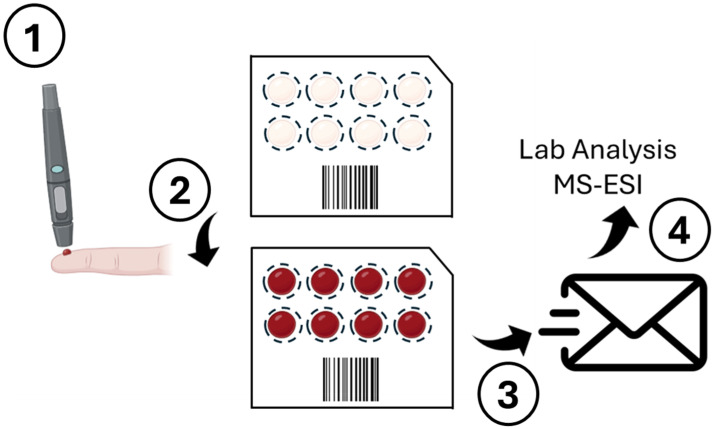
Overview of the DBS sampling process. 1. Blood collection is performed via a fingerstick. 2. The blood is applied onto specialized filter paper. 3. The process advances with the shipment of the dried blood spots to the laboratory. 4. The last step is the subsequent analysis of the samples through MS-ESI. Abbreviations: DBS = dried blood spot; MS-ESI = mass spectrometry–electrospray ionization.

Quantitative analysis has been conducted using LC-MS/MS (liquid chromatography–tandem mass spectrometry), selected for its exceptional precision and specificity in analyte detection. For example, LC-MS/MS allows for the accurate measurement of 3-O-methyldopa (3-OMD) in DBS samples, enabling the diagnosis of aromatic L-amino acid decarboxylase (AADC) deficiency. DBS membrane carriers enable the transmission of samples to diagnostic laboratories for further serological investigation, as well as the long-term storage of biological samples and the creation of biobanks [91]. Figure 6 shows a schematic sampling process of a DBS model.

In clinical practice, DBS is preferable to whole-blood sampling because it lowers the possibility of contamination with infectious and other pathological bioagents [92].

Table 2 summarizes the performances of electrochemical catecholamine sensors based on DBS technology.

## 4. Innovative Nanomaterials in Electrochemical Biosensing for Catecholamines

### 4.1. Polymer Film-Based Electrochemical Sensors

**Figure 7:**
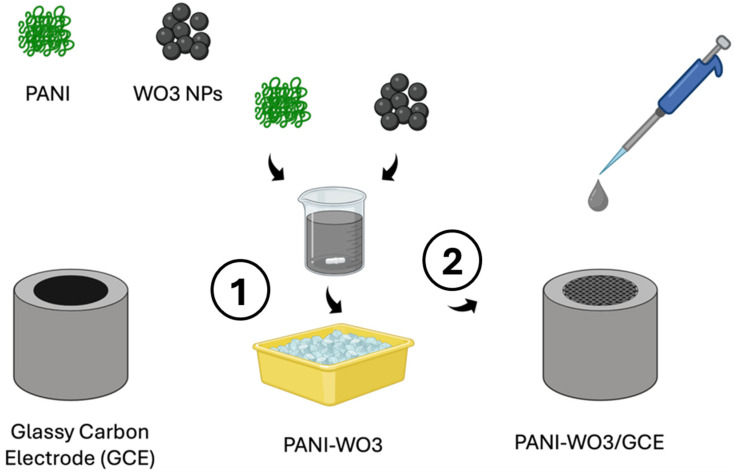
The construction of a polymer-based electrochemical sensor utilizing PANI and WO3 NPs. 1. The process begins with the incorporation of WO3 NPs into the aniline polymerization solution, facilitating the synthesis of a composite material. 2. Subsequently, the PANI-WO3 composite is deposited onto the surface of a GCE, forming the active sensing layer for electrochemical detection. Abbreviations: PANI = polyaniline; NPs = nanoparticles; GCE = glassy carbon electrode; WO3 = tungsten trioxide.

Enhanced sensor performance, in terms of increased conductivity, has been reached through the integration of polymer structures [97]. The process of electropolymerization under galvanostatic, potentiostatic, or, more frequently, potentiodynamic circumstances is the most widely used method for the deposition of polymer films. Polymers provide extremely effective support for the immobilization of biomaterials due to the high degree of chemical and physical stability [98,99]. Figure 7 depicts the construction process of polymer film-based electrochemical sensors built up with a modified glassy carbon electrode (GCE) containing a polyaniline nanocomposite (PANI-WO3).

The analytical methodology developed by Banu, et al. [100] employed a Victoria blue B polymer-modified carbon paste electrode for concurrent differential pulse voltammetry (DPV) measurements of serotonin and epinephrine in the presence of adenine and guanine.

A cutting-edge method was established for dopamine and adrenaline measurement utilizing a single monomer of a carbon black-acryloylated graphene oxide nanocomposite polymer presented by Fatma, et al. [101]. The authors investigated the voltammetric responses in buffer solution, urine, pharmaceutical samples, and human blood and found a detection limit of approximately 0.028 ng mL1 for dopamine and 0.018 ng mL1 for epinephrine. The catecholamine electrochemical biosensor serves as the basis for the application of molecularly imprinted polymer materials (MIPs) for electrode modification [102], known for their portability, affordability, and ease of use [103]. Using poly(3-aminophenylboronic acid) coated via electrochemical polymerization on multi-walled carbon nanotubes (MWCNTs), an MIP synthesis nanocomposite was created for the detection of epinephrine, as proposed by Zhang, et al. [104]. Furthermore, a poly(9-carbazoleacetic acid)-based MIP was used to detect dopamine and epinephrine in plasma samples at the same time [105]. Good sensitivity and detection limit standards were obtained when quantum dots were integrated into an electrode modified with MIPs [106,107]. Table 3 summarizes the performances of electrochemical catecholamine sensors based on polymers.

### 4.2. Carbon Nanomaterial-Based Electrochemical Sensors

Integrating carbonaceous nanoparticles into biosensor transducers significantly enhances signal detection by leveraging their advantageous properties, including high surface-to-volume ratios, chemical stability, excellent conductivity, structural integrity, versatile functionalization potential, and biological compatibility [120,121].

**Figure 8:**
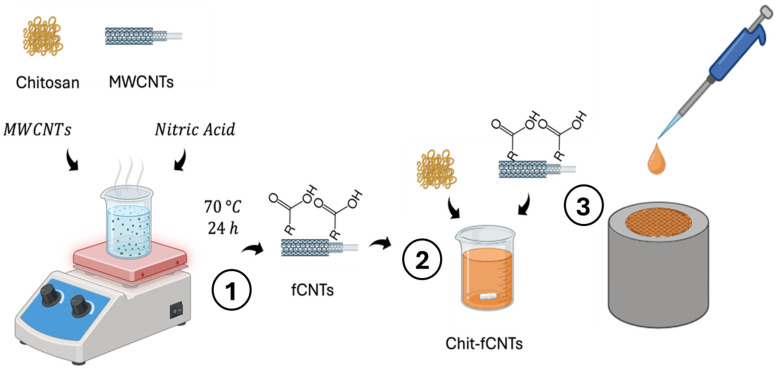
Development of a carbon nanomaterial-based electrochemical sensor utilizing a biocompatible polymer, chitosan, and fCNTs. 1. The process begins with the solution-based synthesis of fCNTs, which are functionalized with carboxylic groups at 70 °C for 24 h. 2. Following synthesis, the fCNTs are dispersed into a chitosan solution to create a composite material. 3. Finally, the chit-fCNTs composite is deposited onto the surface of a GCE, forming the active layer for electrochemical sensing applications. This setup improves sensitivity for detecting specific analytes in biochemical sensing applications. Abbreviations: MWCNTs = multi-walled carbon nanotubes; fCNTs = functionalized multi-walled carbon nanotubes; GCE = glassy carbon electrode.

A new generation of regulated and enhanced electrochemical sensors for ultrasensitive catecholamine detection has been developed taking advantage of the physicochemical properties of graphene, graphene oxide, and carbon nanotubes (CNTs) [122,123]. As shown in Table 3, a very low detection limit in the nanomolar range in biological fluids was attained. In this context, Durairaj, et al. [124] demonstrated the use of MWCNTs blended with Nafion polymer composite-modified carbon electrodes for selective dopamine detection in the presence of uric and ascorbic acids. Indeed, Numan, et al. [125] created a nanocomposite made of MWCNTs and cobalt oxide nanocubes for a highly sensitive platform for amperometric dopamine determination with a detection limit of roughly 0.176 nM. Figure 8 summarizes the working process of an example of carbon nanomaterial-based electrochemical sensors.

Farahani and Sereshti [126] developed an innovative point-of-care device incorporating three-dimensional graphene (3DG) and CNT-modified screen-printed gold electrodes, offering an economical electrochemical sensing platform. The devised portable method detected ascorbic acid, dopamine, and uric acid with detection limits of around 2.5, 0.4, and 0.6 µM, respectively. In a different work, a modified glassy carbon electrode (GCE) with mesoporous carbon and nickel oxide (OMC-NiO) was used to create an electrochemical sensor for the detection of adrenaline [127]. Differential pulse voltammetry was subsequently performed, detecting epinephrine in a linear concentration range of 0.8 µM to 50 µM, with a detection limit of 85 pM and a quantification limit of 0.37 µM.

Kiranmai, et al. [128] demonstrated that combining inorganic particles and reduced graphene oxide (rGO) sheets has the potential to improve sensing performance: a TiO_2_-rGO nanocomposite for epinephrine detection was created using a simple electrospinning process followed by a hydrothermal procedure, with a low detection limit of around 8.11 nM and sensitivity calculated at 0.126 µA/uM.

Joseph, et al. [129] applied a coating of rGO to a carbon paste electrode (CPE). For epinephrine and serotonin, and the redesigned structure achieved a lower limit of detection of around 0.33 and 3.99 nM, respectively, with good sensitivity to real-world sample analysis, high reproducibility, and stability.

Sen, et al. [130] suggested glassy carbon electrode (GCE)-modified graphene oxide sheets and chemically bonded melamine (MGO) for epinephrine detection, unveiling that the electron-rich electron cloud as well as their electron-deficient portions aided the redox reaction. Following modification, the electrode displayed satisfactory sensitivity in the concentration range of 100–600 µM, with a limit of detection of 0.13 µM. Suriyaprakash, et al. [131] developed a flexible point-of-care device for epinephrine monitoring using rGO, achieving a low detection limit of 20 pM with a rapid readout of 2.2 s. The biosensor showed a 60-day lifespan and 95% stability over 25 cycles.

Thondaiman, et al. [132] developed an amperometry technique for simultaneously estimating dopamine and epinephrine. Through chemical oxidation and electrodeposition, they developed a surface-modified copper mesh electrode using a heteroatom-doped Graphene Quantum Dots (GQD)-assisted polymer (PEDOT). The detection limits were around 0.27 µM for dopamine and 0.084 µM for epinephrine. Buleandră, et al. [133] overcame the prior limitation by presenting a two-in-one method for detecting epinephrine and norepinephrine utilizing an electrochemically activated pencil graphite electrode. Epinephrine and norepinephrine peak currents rose with concentrations ranging from 2.5 µM to 250 µM in a combination of analytes. Norepinephrine levels in human plasma samples were 0.83 µM and epinephrine levels were 0.99 µM.

Two alternative techniques have been proposed for dopamine detection: reduced graphene oxide and p-aminophenol [134] and boron-doped nanowalls with electropolymerized polydopamine/polyzwitterion [135].

Luhana, et al. [136] designed a technique using aminated graphene quantum dots (IPAAmGQD) and carboxylic acid cobalt phthalocyanines (CoTCPhOPc) linked to a gold electrode. Dopamine produced the highest current peak, likely due to greater adsorption at the electrode surface. Carbon nanomaterials have proven effective for detecting catecholamines individually and simultaneously in various biological contexts.

A fascinating electrochemical sensor based on carbon nanomaterials was introduced by Li, et al. [137] to real track the quantity of dopamine emitted by living PC12 cells in real time. Furthermore, integrated nanobiosensors based on carbon nanotubes were used to detect the presence of epinephrine in ex vivo rat tissue [138]. Verde, et al. [139] presented an organ-on-screen-printed approach for tracking dopamine release in mouse brains based on a flexible screen-printed substrate. The printed strip yielded a limit of detection of roughly 1 µM and a linear range of up to 160 µM after optimizing the experimental conditions in both buffer and PC-12 cell culture media. Table 4 summarizes the performances of electrochemical catecholamine sensors based on carbon nanomaterials and their derivatives.

### 4.3. Metal Nanoparticle-Based Sensors

**Figure 9:**
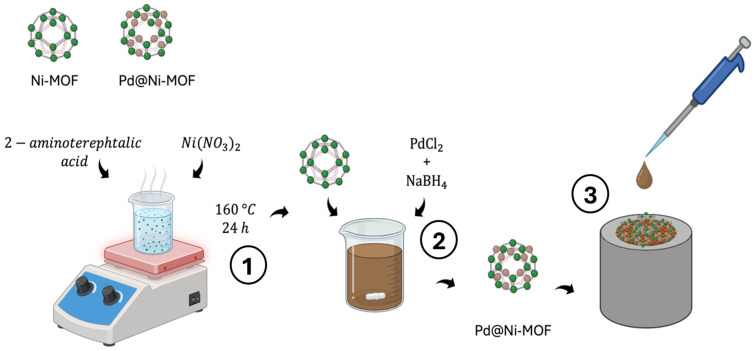
Fabrication of a metal nanoparticle-based electrochemical sensor utilizing Pd and Ni-MOF. 1. The process begins with the solvothermal synthesis of amine-terminated Ni-MOF using 2-aminoterephthalic acid. Ni(NO_3_)_2_ is added to the solution, which is then heated at 160 °C for 24 h. 2. PdCl_2_ and NaBH_4_ are dispersed into the Ni-MOF nanocomposite, which acts as a structural and conductive scaffold for the sensor to facilitate the reduction and incorporation of Pd nanoparticles. 3. Finally, the Pd@Ni-MOF composite is deposited onto the surface of a GCE, creating the active sensing layer for electrochemical detection. Abbreviations: Pd = palladium; Ni-MOF = nickel-based metal–organic framework; PdCl_2_ = palladium chloride; NaBH_4_ = sodium borohydride; GCE = glassy carbon electrode; Ni(NO_3_)_2_ = nickel nitrate.

Several studies suggested immobilizing AuNPs on the electrode surfaces to improve the electrical signals of catecholamine electrochemical detection because of the easy preparation of noble metal nanoparticles and their high surface-to-volume ratio, electrocatalytic ability, and chemical stability [167,168]. Dopamine electrocatalytic redox activity was increased by utilizing gold nanoparticles that were systematically coated with Fe_3_O_4_ magnetic nanocomposites to achieve a detection limit of 2.7 nM [169]. Lim and Zhang [170] demonstrated that combining AuNPs with a multilayer carbon nanomaterial (CNT) produced high-performance electrochemical sensing capabilities and good biostability. Figure 9 depicts the fabrication process of metal nanoparticle-based sensors.

Interestingly, Zhan, et al. [171] modified a free-standing acupuncture needle microelectrode for epinephrine detection using gold nanoparticles and polydopamine. The created probe successfully assessed epinephrine in actual human serum and released by PC12 cells in real time. Table 5 summarizes the performances of metal and metal oxide nanoparticle (NP)-modified electrode surfaces for catecholamine detection.

### 4.4. Enzyme-Based Catecholamine Biosensors

**Figure 10:**
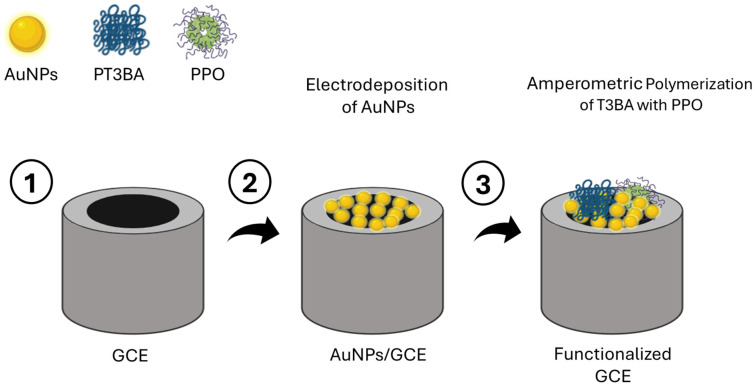
Stepwise fabrication of an electrochemical enzyme biosensor. 1. First, the polishing of the GCE ensures a smooth and clean surface. 2. Then, AuNPs are electrodeposited onto the GCE using the amperometry technique, enhancing the electrode’s surface properties. 3. Finally, the amperometric polymerization of T3BA with PPO occurs on the surface of the AuNPs/GCE, forming a functional layer that facilitates enzyme immobilization and enhances biosensor performance. Abbreviations: AuNPs = gold nanoparticles; PT3BA = poly(3-thiophene boronic acid); PPO = polyphenol oxidase.

Physical adsorption, covalent bonding, integration into a polymer matrix, and cross-linking are the four types of enzyme biomodification approaches [188]. The biocatalytic activity of enzymes is highly dependent on the applied voltage, temperature, and pH of the sample solution. The best reproducibility and sensitivity should be attained under ideal conditions of pH and temperature [189]. The most common enzyme that can use an electrochemical or optical signaling method is tyrosinase. Sethuraman, et al. [190] used AuNPs and poly (thiophene-3-boronic acid) with tyrosinase enzyme (PPO) to measure dopamine levels. The biosensor performance was assessed using the differential pulse voltammetry (DPV) approach. A wide linear detection range of 50 nM to 30 nM with a limit of detection of roughly 20 nM was found. Figure 10 summarizes the fabrication of an enzyme-based biosensor.

The tyrosinase enzyme has recently been mounted on the surface of an AuNP and La_2_O_3_ nanostructured modified indium-tin-oxide electrode by Srivastava, et al. [191]. Long-term stability and reproducibility, as well as a response time of less than 30 s, were attained when compared to the previous approach. The determined detection limit was in the micromolar range (0.258 µM. Similarly, Wu, et al. [192] developed an innovative biosensor for dopamine detection combining the biocatalytic properties of the laccase enzyme and the superior conductivity of carbon quantum dots. The designed enzymatic biosensor achieved a low detection limit of around 80 nM and a broad linear range of 0.25 M to 76.81 M. The catalytic activity of a single-atom ruthenium-based biomimetic enzyme for the simultaneous detection of dopamine and ascorbic acid in real biological serum samples was examined by Xie, et al. [193], proving comparatively low detection limits of 20 nM and 170 nM, respectively. The laccase enzyme immobilized by zwitterionic surfactants was recently immobilized via physical adsorption on the surface of halloysite nanotubes (HNTs) by Decarli, et al. [194]. Using tubular nanomaterials (HNTs) as a scaffold for a sensitive film, a linear calibration plot between dopamine concentrations and peak current was found, with the limit of detection set to 0.252 µM. Following that, a biosensor based on the physical adsorption of MWCNTs on the surface of the electrode, followed by the immobilization of the tyrosinase enzyme, was disclosed [195]. The authors focused on the electrochemical redox mechanism of epinephrine for the scope, and two reduction peaks were seen during the reduction process at 0.181 V and 0.229 V. The proportionality of the reduction currents with increasing epinephrine concentration was demonstrated by DPV measurements of epinephrine at various concentrations, finding a solid linear relationship between peak currents and epinephrine concentrations and an acceptable limit of detection of 0.51 µM.

Table 6 summarizes the performances of electrochemical catecholamine enzymes based on biosensors.

### 4.5. DNA Aptamer-Based Catecholamine Biosensors

An affinity ligand is essential for achieving selectivity; however, electrodes functionalized with the aforementioned nanomaterials provide the advantage of an expanded surface area. Additionally, aptamers have garnered significant attention as recognition elements in biosensing design due to their affordability, compact size, and exceptional chemical stability [201]. Thanks to the exceptional capacity to identify and attach to target molecules, aptamer technology can address difficulties of low selectivity, limit interference from other molecules, and demonstrate exceptional structural flexibility. Aptamer-based proposals for catecholamine detection and relative performances are listed in Table 7. Zhang, et al. [202] enhanced the performance of a dopamine aptasensor using a modified cerium–metal framework (Ce-MOF). A single-stranded nucleic acid (S1) was paired with the aptamer to form a double strand and linked to a Ce-MOF-modified electrode, while a second sequence (S2) was attached to the signal material to create a probe. The resulting biosensor demonstrated a strong affinity for dopamine detection with a detection limit of around 6 pmol/L.

**Figure 11:**
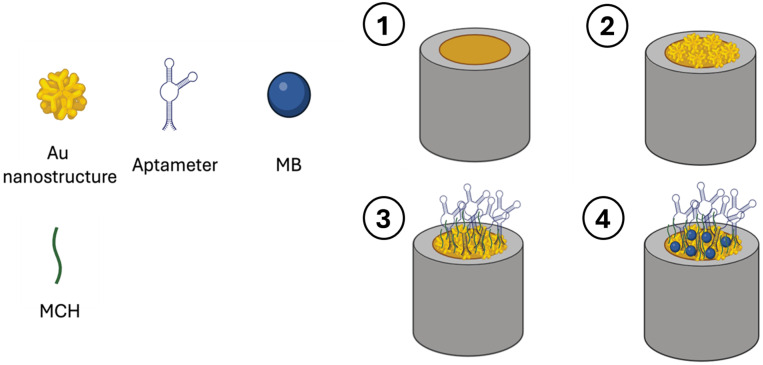
Steps involved in the fabrication of an electrochemical aptasensor. (1) The process begins by polishing the Au electrode to achieve a smooth and clean surface. (2) Then, spindle-shaped gold nanostructures are electrodeposited onto the Au electrode using the chronoamperometry technique, enhancing the electrode’s surface area and properties. (3) A thiolated RNA aptamer with dopamine binding sites is then deposited onto the surface, with MCH employed to minimize nonspecific attachment of aptamer molecules. (4) Finally, MB is deposited to further enhance electron transfer, optimizing the sensor’s electrochemical response. Abbreviations: Au = gold; MB = methylene blue; MCH = mercaptohexanol.

Most aptamer-based electrochemical biosensors have only been tested in buffer solutions and seem most suitable for ex vivo studies, in which biological materials can be processed for optimal sensor performance. Furthermore, putative unspecific interactions between target molecules and AuNP surfaces are frequently overlooked, resulting in misread findings. Figure 11 shows the steps involved in the fabrication process of an aptamer-based electrochemical sensor.

In order to address this issue, Wu, et al. [203] demonstrated that the implantable aptamer–graphene field-effect transistor (G-FET) probe could be used to monitor dopamine release in vivo in mice models in real time. The designed biosensor demonstrated both excellent selectivity and picomolar sensitivity. Later, the same soft neural probe-based method was used to detect multi-neurotransmitters via electrografting-enabled site-selective functionalization of aptamers on G-FETs. COOH and NH2 electrografted functional groups served as linkers to functionalize two aptamers with different functional groups (NH2 and COOH). When dopamine or serotonin binds to its specific aptamer, the functionalized aptamer on the graphene undergoes a conformational shift, which modifies the doping state of the graphene and results in a current change on the source drain of the GFET probe. Despite the low detection limit and good selectivity, aptamer-based biosensor technology still needs further development due to its complex chemistry.

**Table 7:** Performances of electrochemical catecholamine sensors based on DNA aptamer technologies.

Molecule	Electrode Modification	Interferents	Technique	Linear Range	Detection Limit	Ref
EP	Aptamer based Organic electrochemical transistors	DA, Cysteine, AA, tryptophan	Amperometry	0.0009–90 µM	0.9 nM	[204]
DA	Aptamer-AuNPs-rGO/GCE	AA, UA, EP, and cathechol	DPV	0.001–0.1 µM	47 nM	[205]
DA	Aptamer-Copper aluminate-rGO-TEPA/SPE	UA, AA, and glucose	DPV	0.00005–10 µM	0.017 nM	[206]
DA	Aptamer-CeMOF/GCE	AA, BSA, and bilirubin	SWV	0.0005–0.1 µM	0.06 nM	[202]
DA	Aptamer-GCSC-GO/GCE	DOPA, AA, HVA	DPV	0.001–1 µM	0.75 nM	[207]
DA	Aptmer-Gold nanostructure/Au electrode	AA, UA, Catechol, EP, and NE	DPV	0.000163–0.02 µM	0.01 nM	[208]
EP	Aptamer-Methylene blue/GCE	AA, UA, and levodopa	CV, DPV	0.2–10 µM	67 nM	[209]

Abbreviations: DA = dopamine; EP = epinephrine; TEPA = tetraethy lenepentamine; CeMOF = Cerium-based metal–organic framework; GCSC-GO = grass carp skin collagen-graphene oxide; AuNPs = gold nanoparticles; rGO = reduced graphene oxide.; DPV = differential pulse voltammetry; CV = cyclic voltammetry; SWV = squarewave voltammetry; AA = ascorbic acid; UA = uric acid; BSA = bovine serum albumin, HVA = homovanilic acid.

## 5. Clinical Implications

From a clinical standpoint, fluctuations in plasma levodopa concentrations are mirrored by corresponding changes in striatal dopamine levels, leading to non-physiological, pulsatile stimulation of dopamine receptors in advanced PD patients. Plasma levodopa levels have been shown to reliably predict the onset of ON–OFF transitions, as well as the emergence of motor fluctuations and LID onset. [210,211,212]. However, the current standard of motor assessment still relies on clinical evaluation, with a lack of reliable tools for fine-tuning therapeutic interventions based on real-time levodopa levels.

**Figure 12:**
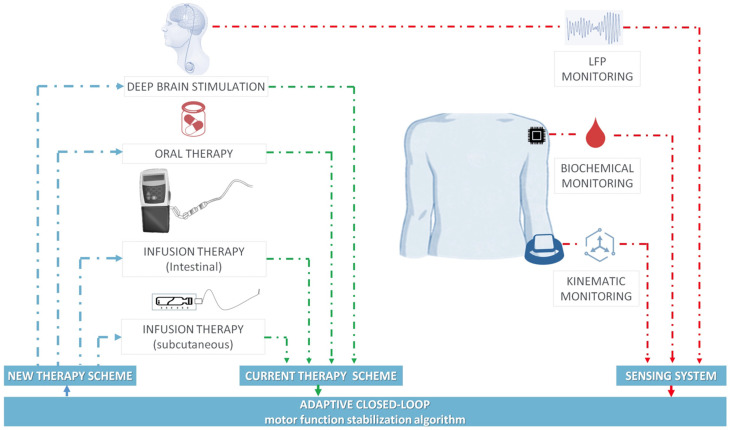
Multimodal adaptive closed-loop therapy system in advanced PD. Advanced therapeutic strategies—such as deep brain stimulation, oral medications, intestinal infusion therapy, and subcutaneous infusion therapy, used individually or in combination—can be delivered via an adaptive closed-loop framework. This system incorporates a modular, multiparametric sensing platform capable of monitoring local field potentials, biochemical markers, and kinematic data for personalized therapy optimization. Reproduced, under the terms of the Creative Commons Attribution 4.0 License, from [43].

Nonetheless, the fine estimation of plasma levodopa levels in routine practice still remains an unmet need. For this purpose, augmenting motor performance assessment (e.g., through levodopa monitoring) should be preliminary in the path toward a self-regulating adaptive system for PD management [213], as shown in Figure 12.

Biochemical, neurophysiological, and wearable sensors represent key components in providing feedback signals to close the analysis-modulation loop. This adaptive therapy paradigm marks a significant advancement over traditional open-loop approaches, which rely on fixed treatment schemes that cannot adjust to real-time fluctuations. From a neurophysiological perspective, synchronized oscillatory rhythms consistently linked to bradykinetic and dyskinetic states can serve as effective control signals for closed-loop deep brain stimulation (DBS) [214]. Subcortical sensing of neuronal firing activity, combined with the real-time extraction of symptom correlates during DBS, represents the most refined closed-loop strategy [11]: Bradykinesia is driven by basal ganglia activity in the 13–30 Hz range (β frequency), which remains the most promising neurophysiological marker for guiding closed-loop DBS in PD [215,216,217]. In contrast, basal ganglia activity in the γ range (25–140 Hz) serves as the neurophysiological hallmark of LIDs. On the inertial side, wearable sensors paired with machine learning algorithms can transform kinematic motor data into quantitative signals, enabling the recognition and prediction of motor state changes [218]. In this regard, Rojas, et al. [219] compared acceleration data from Medtronic’s RC + S device and Apple Watches in PD patients during home use. Spectral analysis showed the external accelerometer had higher power in the Parkinsonian tremor band (4–7 Hz), while the DBS electrodes had more power in higher frequencies (7–30 Hz), suggesting that the accelerometer could be useful for assessing tremor and motor states in closed-loop DBS. Notably, microtablets of levodopa/carbidopa (Flexilev®, Flexilev, Oslo, Norway) can optimize levodopa administration through a wearable sensing system (e.g., PKGä) allowing a more precise drug dosing with a minimum of 5 mg of levodopa [220]. Indeed, an algorithm used by Pulliam, et al. [221] with the Kinesia^ä^ system (Great Lakes NeuroTechnologies, Cleveland, OH, USA) has been proven to provide a quantitative evaluation of motor symptoms and the transition of medication states: the algorithm was compared to clinician video assessment and showed an area under the curve (AUC) of 0.89 for tremor, 0.86 for dyskinesia, and 0.82 for bradykinesia, with high sensitivity and specificity. Wearables have also been used to predict responses to levodopa as a supporting tool for either the clinical diagnosis of PD or the suitability of advanced therapies in fluctuating PD patients. Finally, an ML model from Khodakarami, et al. [222] demonstrated that inertial data from PKG^ä^ could replicate the levodopa challenge test response with a maximum AUC of 0.89.

Biochemical sensing of levodopa and its metabolites plays a pivotal role in enabling multimodal closed-loop therapy for Parkinson’s Disease. This approach holds significant promise for patients receiving oral or infusion therapies as it facilitates the monitoring of both gradual pharmacodynamic changes linked to disease progression [12,14,72] and faster dynamics, such as motor fluctuations and dyskinesias [210,212].

In this regard, non-invasive methods, such as sensors based on sweat analysis, offer the advantage of ease of use and patient comfort but often suffer from lower accuracy and a limited detection range compared to invasive techniques. Microdialysis and interstitial fluid sensors provide higher accuracy and temporal resolution, but their invasiveness makes them less suitable for continuous long-term monitoring in clinical practice. Nanomaterial-based sensors, including carbon nanotubes and metal–organic frameworks (MOFs), have demonstrated a wide range of applications in electrochemical biosensing. These materials have emerged as leading candidates for electrode modification due to their exceptional ability to enhance sensitivity and selectivity, achieving detection limits as low as 0.45 μM. Their remarkable properties, such as unique dimensions and morphologies, have made them invaluable in research areas like biological molecule immobilization, catalysis of electrochemical reactions, acceleration of electron transfer, and the overall enhancement of sensor performance.

While promising, several limitations must be addressed before biochemical sensing technologies can transition from laboratory-based findings to routine clinical applications. One significant translational challenge lies in bridging the gap between proof-of-concept demonstrations and scalable, real-world solutions. Additionally, issues related to selectivity, sensitivity, and interference from other biological compounds remain key barriers to widespread clinical use. For example, the simultaneous detection of multiple catecholamines is achievable in controlled laboratory conditions, but maintaining this selectivity in the complex in vivo environment, where numerous metabolites and proteins can interfere, remains a challenge. Overall, while these technologies show great promise for real-time, personalized therapy management in PD, further improvements in wearability, miniaturization, and reliability are needed before they can be routinely adopted in clinical settings.

PD exhibits significant inter- and intra-patient variability in symptom expression, encompassing both motor and non-motor domains. While motor symptoms are traditionally the primary focus of PD assessment, non-motor symptoms—such as sleep disturbances, autonomic dysfunction, and cognitive decline—often have a greater impact on a patient’s quality of life than motor symptoms. These non-motor features, now widely acknowledged as core components of PD, are frequently underrecognized in conventional clinical assessments [223]. Alterations in sleep architecture and disruptions in heart rate variability may indicate underlying autonomic dysfunction, revealing fluctuations that might otherwise remain undetected during standard examinations or motor-centered diaries [223]. There is an urgent need for unobtrusive, multimodal platforms capable of capturing real-time fluctuations in levodopa levels and non-motor symptoms within home settings. Continuous monitoring of these non-motor symptoms in real-world environments, rather than relying solely on patient recall during clinical visits, significantly enhances therapeutic outcomes. Integrating sensor-derived data to track the full spectrum of symptoms alongside levodopa concentrations provides a more holistic view of the disease. Moreover, recent advancements in telemedicine have underscored the value of incorporating emerging non-invasive technologies, such as radio frequency (RF) sensors operating in the ultra-wideband (UWB) spectrum, into existing monitoring systems. These innovations enable continuous, objective, and unobtrusive assessments of motor performance, daily activities, and vital signs [224,225]. Such platforms would provide a holistic view of disease progression and response to therapy, moving beyond the current focus on motor symptoms alone [41,223].

Furthermore, from a technological standpoint, the routine implementation of biochemical sensing is hindered by the absence of fully wearable, standalone sensing systems that are both patient-friendly and durable for extended use. Furthermore, the lack of validated algorithms capable of accurately correlating fluctuations in the blood levels of levodopa and its metabolites with motor symptoms remains a critical limitation. In this regard, despite current neuro-computational models being capable of predicting both brain levodopa concentrations as a function of plasma concentrations and the resulting temporal patterns of finger-tapping frequency [13], peripheral (plasma) dosing may not be sufficiently precise, and direct brain-level monitoring remains essential to accurately capture the biochemical–motor relationship. These algorithms must also incorporate a detailed understanding of the BBB transport kinetics, which play a pivotal role in determining central levodopa bioavailability and therapeutic efficacy.

In terms of technology readiness levels (TRLs), biochemical sensing for closed-loop therapy in PD remains between TRL 3 (Proof-of-Concept Demonstrated) and TRL 4 (Technology Validated in Laboratory), highlighting the need for further development, including miniaturization, wearability, and reliability enhancements. Finally, broader methodological considerations, such as the standardization of data collection protocols, cross-platform validation, and addressing patient usability concerns, are essential to ensure the successful translation of these promising technologies into routine clinical practice.

## 6. Conclusions

The objective monitoring of motor symptoms is still an unmet need in PD and is crucial for optimizing therapeutic management, particularly in advanced disease stages where patients experience motor fluctuations and dyskinesias. The current lack of quantitative biomarkers for continuous assessment underscores the need for advanced sensing technologies to guide more precise and personalized therapeutic interventions. Biochemical sensing holds significant promise for advancing personalized therapy in PD. Despite the current limitations in wearability, invasiveness, and technology readiness levels, these sensors offer a pathway for monitoring levodopa and other catecholamines, potentially augmenting real-time motor performance assessment and therapy adjustments. With further advancements in sensor miniaturization, reliability, and selectivity, biochemical sensing may soon play a key role in closed-loop systems for more effective and individualized PD management.

## Figures and Tables

**Table 1:** Performances of wearable bio-sensors for catecholamine detection.

Molecule	Sample	Electrode Modification	Technique	Sensitivity	Linear Range	Detection Limit	Ref.
LD	Sweat	Nafion 117/Tyr + Gluteraldehyde/Polythionine/Au Nanodendrites/Au/Cr	CV	1.7 nA/μM	1.25–20 μM	1.25 μM	[68]
LD	Sweat	Hydrogel/Tyr + Gluterladehyde/SPCE	Chronoamperometry	NA	5–30 μM	300 μM	[74]
LD	ISF	PU Layer/PANI-Enzyme Layer (Tyr)/Nafion/Au Nanodendrites/Stainless Steel MNs	Chronoamperometry	12.6 nA/μM	0–20 μM	0.18 μM	[77]
EP	Sweat	PANi-co-PBA/AuNPs/SPCE	DPV	NA	NA	0.60 nM	[80]
DA	Sweat	ACC-α-Fe_2_O_3_	CV	NA	0.074–113 μM	50 nM	[76]
DA	Sweat	MWCNT-COOH/SPCE	CV	NA	0–70 µM	0.043 µM	[69]
LD	ISF	Microneedle/spike- like Au Nanoparticles	Chronoamperometry	12.6 nA/μM	NA	0.18 μM	[81]
LD	ISF	CP + Tyr/MNs	SWV, Chronoamperometry	0.037 μA/μM	5–100 μM, 100–300 μM	0.5 μM	[78]
LD	Sweat	Au/ZIF-8/GO@Tyr	Chronoamperometry	NA	1–95 μM	0.45 μM	[75]
LD	ISF	Methacrylated hyaluronic acid microneedle/Au sputtered interdigitated electrode array	CV, Chronoamperometry	NA	1–1000 μM	1 μM	[79]
DA, NEP, EP	Artificial ISF	h-nPG	CV	2.4 ± 0.05 µA/µM	5–850 µM	0.1 µM	[82]
DA	ISF	N-UNCD/TiE	CV	NA	1.0–30 µM	NA	[83]
LD	Sweat	Tyr@ZIF-8/GO/SPCE	Chronoamperometry	0.0073 µA/µM	0–100 µM	0.28 µM	[84]
LD	Sweat	3DpCE	DPV	83 ± 3 nA/μM	300 nM–24 µM	30 nM	[85]
EP, DA	ISF	(Dopamine-imprinted PANI-co-PBA) and Epinephrine-imprinted PANI-co-PBA	CV	NA	0.304–121 μM; 0–763 nM	0.0007–2.11 nM	[86]

Abbreviations: NA = not available; LD = levodopa; DA = dopamine; NEP = norepinephrine; EP = epinephrine; MNs = microneedles; SPCE = screen-printed carbon electrode; PU = polyurethane; PANi = polyaniline; ACC = acid-treated carbon cloth; PBA = phenylboronic acid; ZIF-8 = zeolitic imidazolate framework; GO = graphene oxide; MWCNT-COOH = multi-walled carbon nanotubes carboxylated; Tyr = tyrosinase; h-nPG = highly nanoporous gold; N-UNCD = nitrogen-incorporated ultrananocrystalline diamond; Tie = titanium electrode; 3DpCE = 3D-printed carbon electrodes; CP = carbon paste; DPV = differential pulse voltammetry; CV = cyclic voltammetry.

**Table 2:** Performances of electrochemical catecholamine sensors based on DBS technology.

Molecule	Instrumentation	Linear Range	Detection Limit	Ref.
3-OMD; 5-HTP	MS-ESI	25–5000 nM	2.4 nM; 1.4 nM	[93]
3-OMD	LC–MS/MS	0–4280 nM	94 nM	[94]
3-OMD	HPLC-MS/MS method	839 to 5170 ng/mL	NA	[95]
3-OMD	MS-ESI	0.31–4.6 µM	0.24 µM	[96]

Abbreviations: NA = not available; 3-OMD = 3-O-methyldopa; 5-HTP = 5-Hydroxytryptophan; MS-ESI: mass spectrometry–electrospray ionization; HPLC-MS = high-performance liquid chromatography–mass spectroscopy; LC-MS/MS: liquid chromatography–tandem mass spectroscopy.

**Table 3:** Performances of electrochemical catecholamine sensors based on polymers.

Molecule	Catalyst	Technique	Linear Range	Detection Limit	Ref.
DA	Poly paraphenylene diamine/GCE	DPV	0.038–4.76 µM	0.094 nM	[108]
DA	PPy matriNA supported iron/GCE	CV	10–900 µM	321–348 nM	[109]
DA	PANi-WO3/GCE	CV, DPV	20–300 µM	139 nM	[110]
DA	Polymelamine-AuNPs/CPE	CV, DPV	0.2–11 µM	67 nM	[111]
DA	PPyoNA/LSGE	CV, DPV	0.010–10 µM	7 nM	[112]
EP, 5-HT	Poly Victoria blue B/CPE	DPV	1.0–80 µM	330–980 nM	[100]
DA	Poly-tryptophan/GCE	DPV	0.2–100 µM	60 nM	[113]
DA	Copper monoamino-phthalocyanine-acrylate polymer/GCE	DPV	0.01–10 µM	0.7 nM	[114]
NEP	Ag NPs@N/GQDs	DPV	0.5–700 pM	0.154 pM	[115]
DA	DA imprinted PPy-ta-C/CNFs	DPV	0.01–10 µM	5.4 nM	[116]
DA	DA imprinted PPy/MWCNT/GAs (multi-walled carbon nanotubes spaced graphene aerogels (MWCNT/GAs))	DPV	0.005–20 µM	1.67 nM	[117]
DA	DA imprinted PPy-o-PD(o-phenylenediamine)	DPV	3–100 µM	3972 nM	[118]
DA	DA imprinted PPy/pThi/NPGc (Poly thionine nanoporous gold)	DPV	0.05–0.2 µM	100 nM	[119]

Abbreviations: DA = dopamine; NEP = norepinephrine; EP = epinephrine; GCE = glassy carbon electrode; CNFs = carbon nanofibers; LSGE = laser-scribed graphene electrode; CPE = carbon paste electrode; GQDs = graphene quantum dots; PANi = polyaniline; PPy = polypyrrole; PPyox = polypyrrole oxidized; DA-imprinted PPy-ta-C = dopamine-imprinted polypyrrole-terephthalic acid co-polymer; DA-imprinted PPy = dopamine-imprinted polypyrrole; MWCNT/Gas = multi-walled carbon nanotubes spaced graphene aerogels; o-PD = o-phenylenediamine; pThi = poly thionine; NPGc = nanoporous gold; PPyox = overoxidized polypyrrole; AgNPs = silver nanoparticles; o-PD = o-phenylenediamine; DPV = differential pulse voltammetry; CV = cyclic voltammetry.

**Table 4:** Performances of electrochemical catecholamine sensors based on carbon nanomaterials.

Molecule	Sample	Electrode Modification	Technique	Sensitivity	Linear Range	Detection Limit	Ref.
DA	Human blood serum	MWCNTs-ZnO/GCE	CV, DPV	16 µA/µM	0.01–30 µM	3.2 nM	[140]
DA, 5-HT	PBS	Curcumin oNAidized carbon nanotubes/GCE	LSV	NA	0–170 10–130 µM	0.010–0.011 nM	[141]
EP	Real water	MWCNTs-molybdenum disulphide/GCE	CV	NA	9.9–137.9 µM	0.003 nM	[142]
DA, EP	Synthetic urine	ONAidized capsaicin-MWCNTs/GCE	CV Amperometry	DA 12 nA/µM EP 3 nA/µM	5–75 5–115 µM	0.0072–0.0015 nM	[143]
EP	Urine and pharmaceutical sample	Chitosan-functionalized carbon nanotubes/GCE	CV, DPV	NA	0.05–10 µM	30 nM	[144]
DA	Human blood serum	CaCO_3_-PANi-rGO/GCE	DPV	NA	0.1–14 µM	100 nM	[145]
DA, UA	PBS	Thermally rGO/GCE	CV, DPV	28.64 µA/µM	5.0–42 µM	120–150 nM	[146]
DA	Human urine	rGO-tungsten trioNAide/GCE	CV, Amperometry	0.392 µA/µM	0.3–1245 µM	306 nM	[147]
EP	PBS	rGO-MoS_2_-Fe_3_O_4_/GCE	CV, DPV	2.87 µA/µM	0–11 µM	137 nM	[148]
EP	Human blood serum	2D nickel oNAide-rGO/GCE	CV, DPV	NA	50–500 µM	1000 nM	[149]
EP	Human urine	rGO-Ti_3_ C2 TNA MNAene/ITO	DPV	3.74 µA/µM	1.0–60 µM	3.5 nM	[150]
DA	PBS	GO-CuAIO_2_/GCE	LSV	NA	0.92–10 µM	15 nM	[151]
EP	Human blood serum	Au-Pd-rGO/GCE	CV, DPV	NA	0.001–1000 µM	12 nM	[152]
NEP	Artificial Sweat	ECR/GCE	DPV	NA	2.0–50 µM	1.5 µM	[153]
NEP	Synthetic Urine, Human Urine	MCM-41/CPE (Mesoporous Silica)	DPV	NA	0.07–2000 µM	0.04 µM	[154]
DA	Human Urine	CeO_2_-PEDOT/MWCNT/GCE	DPV	NA	0.1–10 µM	0.03 µM	[155]
DA	PBS	RuS_2_ NPs/GCE	Chronoamperometry	1.8 µA/µM	0.1–10 µM	0.074 µM	[156]
LD	Urine	PGSSG/MWCNTs/GCE (electropolymerizing glutathione disulfide (PGSSG)	DPV	NA	1–1000 μM	0.33 μM	[157]
LD	Human serum	Chitosan/Au NPs/SWCNTs/CPE	DPV	NA	0.5–200 μM	0.5 μM	[158]
LD	Human Serum, Urine	GO/ZnO/CPE	SWV	NA	1–1000 μM, 1–800 μM	0.36 μM	[159]
LD	Human Serum, Urine	AuNP-CNTs/PGE	CV	NA	0.1–150 μM	50 nM	[160]
LD	Urine	3D GF/ITO	CV	0.24 μA/μM	1–60 μM	1 μM	[161]
DA	PBS	nPG/GCE	CV	NA	0–14 µM	17 nM	[162]
DA	PBS	nPG/AuE	DPV	NA	0.1–40 µM	100 nM	[163]
LD	PBS	In2S3NSPs/3DGr/ITO electrode	CV	1.98 μA/μM	0–60 μM	87 nM	[164]
EP	PBS	Gold nanoporous film modified AuE	CV	NA	50–1000 µM	19 µM	[165]
LD	Human serum	Nafion/Tyr + BSA + Gluteraldehyde/GO/GCE	Chronoamperometry, CV	NA	0–200 µM	0.84 µM	[166]

Abbreviations: NA = not available; LD = levodopa; DA = dopamine; NEP = norepinephrine; EP = epinephrine; UA = uric acid; GCE = glassy carbon electrode; CPE = carbon paste electrode; PGE = pencile graphite electrode; ZnO = zinc oxide; rGO = reduced graphene oxide; GO = graphene oxide; ECR = Eriochrome Cyanine R; MCM-41 = mesoporous silica; MWCNTs = multi-walled carbon nanotubes; PGSSG = electropolymerizing glutathione disulphide; nPG = nanoporous gold; 3D GF = 3D porous graphene foam; ITO = indium tin oxide; AuE = gold electrode; PANi = polyaniline; AuNp = gold nanoparticles; CNTs = carbon nanotubes; 3DGr = 3D graphene; SWCNTs = single-walled carbon nanotubes; BSA = bovine serum albumin; DPV = differential pulse voltammetry; CV = cyclic voltammetry; LSV = linear sweep voltammetry; SWV = squarewave voltammetry.

**Table 5:** Performances of electrochemical catecholamine sensors based on metal and metal oxide NPs.

Molecule	Electrode Modification	Technique	Sensitivity	Linear Range	Detection Limit	Ref.
DA	Au/Ti_3_C_2_TNA/GCE	CV, DPV	0.0925 μA/μM	10–100 µM	15 µM	[172]
DA	Citrate-stabilized gold nanoparticles @polydopamine)/PGE	SWV	NA	0.5–7.0 µM	0.53 µM	[173]
DA	Copper nanoparticles/GCE	CV, DPV	NA	0.05–5.0 µM	0.04 µM	[174]
DA	Carbon quantum dots and copper oNAide/GCE	SWV	NA	1–180 µM	25.4 µM	[175]
DA	AuNPs and Nafion/Diamond nanoporous	SWV	NA	3–100 µM	0.068 µM	[176]
DA	Gold-decorated porous silicon-poly(3-heNAylthiophene)/GCE	Amperometry	0.5112 μA/μM	1–460 µM	0.63 µM	[177]
DA, UA	Cu-based metal–organic frameworks/CPE	DPV	NA	0.05–500 µM	0.03–0.07 µM	[178]
DA	Copper organic framework@halloysite nanotubes-rGO/GCE	DPV	NA	0.1–130 µM	0.015 µM	[179]
DA	Carbon-titanium nitride nanoparticles/GCE	DPV	9620 µA/µM	0.1–250 µM	0.03 µM	[180]
DA	Palladium nanoparticles decorated nickel-based metal–organic framework/GCE	CV, DPV	NA	0.001–100 µM	0.01 µM	[181]
DA	Nitrogen-doped titanium dioNAide-AgNPs-GQD/GCE	CV, DPV	NA	0.003–300 µM	0.001 µM	[182]
DA	Nanoplatelets of zinc oNAide embedded polyvinyl alcohol/FTO	EIS	NA	0.020–3000 µM	0.005 µM	[183]
DA	Cobalt phthalocyanine-nitrogen-doped GQDs/GCE	Amperometry	7.21 µA/mM	100–1000 µM	0.12 µM	[184]
DA	Sodium tungsten bronzes nanoparticles/Carbon spheres	DPV	19.335 μA/μM	0.004–106.4 µM	0.001 µM	[185]
NE	Graphene quantum dots decorated AuNPs/GCE	DPV	4.2 × 10^−7^ µA/µM	0.5–7.5 µM	0.15 µM	[186]
DA, EP	Nickel telluride/CPE	SWVs	NA	4.0–31 µM	0.15–0.35 µM	[187]

Abbreviations: NA = not available; DA = dopamine; NEP = norepinephrine; EP = epinephrine; UA = uric acid; GQDs = graphene quantum dots; AuNPs = gold nanoparticles; SH = sulfhydryl; CuMOF/CPE = copper-based metal–organic framework/carbon paste electrode; DPV = differential pulse voltammetry; CV = cyclic voltammetry, SWV = squarewave voltammetry; EIS = electrochemical impedance spectroscopy.

**Table 6:** Performances of electrochemical catecholamine enzymes based on biosensors.

Molecule	Electrode Modification	Technique	Sensitivity	Linear Range	Detection Limit	Ref.
DA	PT3BA/Tyr/AuNPs/GCE	DPV	NA	0.5–0.3 µM	0.2 nM	[190]
DA	Tyr/Au-La_2_O_3_/ITO	CV	24.8 µA/µM	2–100 µM	0.258 µM	[191]
DA	Lac/CDs/GCE	EIS, DPV	NA	0.25–76.81 µM	0.08 µM	[192]
DA	Ru-Ala-C_3_N_4_/GCE	CV, DPV, EIS	NA	0.06–490 µM	0.02 µM	[193]
DA	Lac-HNT-ImS3-14/CPE	SWV	NA	0.99–67.8 µM	0.252 µM	[194]
EP	Tyr/MWCNTs/GCE	CV, DPV, EIS	NA	0.6–100 µM	0.51 µM	[195]
DA	d-amino acid oNAidase and hemoglobin/MnO_2_ nanoparticles enriched poly thiophene	CV	12.801 µA/µM	0.04–9.0 µM	41 nM	[196]
DA	Lac/MWCNTs/GCE	SWV	3.64 µA/µM	0.1–10 µM	1.33 µM	[197]
DA	Fe_3_O_4_@SiO_2_/GCE	CV, EIS	NA	1.5–7.5 µM	0.177 µM	[198]
LD	Nafion/Tyr + BSA + Gluteraldehyde/ Polythionine/CNTs/CPE	Chronoamperometry	NA	0.8–22 µM	2.5 µM	[199]
LD	Tyr + BSA/PB Carbon Paste	CV, Amperometry	1.23 nA/µM	0–10 µM	0.25 µM	[200]

Abbreviations: NA = not available; LD = levodopa; DA = dopamine; EP = epinephrine GCE = glassy carbon electrode; CPE = carbon paste electrode; MWCNTs = multi-walled carbon nanotubes; CNTs = carbon nanotubes; PB Carbon Paste = Prussian Blue carbon paste; PT3BA = Poly(thiophene-3-boronic acid); BSA = bovine serum albumin; Tyr = tyrosinase enzyme; ITO = indium tin oxide; AuNPs = gold nanoparticles; Lac = laccase enzyme; CDs = carbon dots; HNT = halloysite nanotube; SWV = squarewave voltammetry.
